# Integrated metasurfaces for re-envisioning a near-future disruptive optical platform

**DOI:** 10.1038/s41377-023-01169-4

**Published:** 2023-06-20

**Authors:** Younghwan Yang, Junhwa Seong, Minseok Choi, Junkyeong Park, Gyeongtae Kim, Hongyoon Kim, Junhyeon Jeong, Chunghwan Jung, Joohoon Kim, Gyoseon Jeon, Kyung-il Lee, Dong Hyun Yoon, Junsuk Rho

**Affiliations:** 1 Department of Mechanical Engineering, Pohang University of Science and Technology (POSTECH), Pohang, 37673 Republic of Korea; 2 Department of Chemical Engineering, Pohang University of Science and Technology (POSTECH), Pohang, 37673 Republic of Korea; 3 Research Institute of Industrial Science and Technology (RIST), Pohang, 37673 Republic of Korea; 4 POSCO-POSTECH-RIST Convergence Research Center for Flat Optics and Metaphotonics, Pohang, 37673 Republic of Korea

**Keywords:** Metamaterials, Nanophotonics and plasmonics, Integrated optics, Displays

## Abstract

Metasurfaces have been continuously garnering attention in both scientific and industrial fields, owing to their unprecedented wavefront manipulation capabilities using arranged subwavelength artificial structures. To date, research has mainly focused on the full control of electromagnetic characteristics, including polarization, phase, amplitude, and even frequencies. Consequently, versatile possibilities of electromagnetic wave control have been achieved, yielding practical optical components such as metalenses, beam-steerers, metaholograms, and sensors. Current research is now focused on integrating the aforementioned metasurfaces with other standard optical components (e.g., light-emitting diodes, charged-coupled devices, micro-electro-mechanical systems, liquid crystals, heaters, refractive optical elements, planar waveguides, optical fibers, etc.) for commercialization with miniaturization trends of optical devices. Herein, this review describes and classifies metasurface-integrated optical components, and subsequently discusses their promising applications with metasurface-integrated optical platforms including those of augmented/virtual reality, light detection and ranging, and sensors. In conclusion, this review presents several challenges and prospects that are prevalent in the field in order to accelerate the commercialization of metasurfaces-integrated optical platforms.

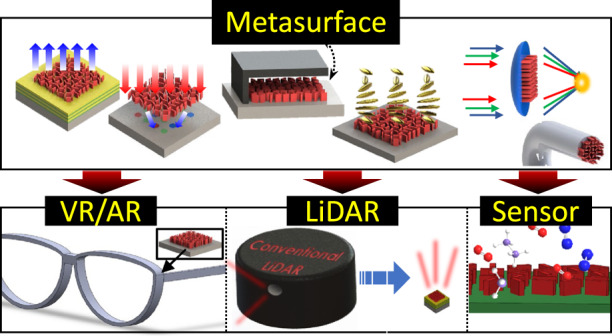

## Abstract (web summary)

We organize integrated metasurfaces with conventional devices (LED, LC, MEMS, etc.), and their potential applications in VR/AR, LiDAR, and sensors.

## Introduction

Metasurfaces, two-dimensional (2D) arrays of subwavelength artificial structures (also referred to as meta-atoms), have emerged as alternatives to conventional refractive optical elements (ROEs) and diffractive optical elements (DOEs). They exhibit the ability to construct compact form factors with arbitrary manipulation of outgoing light^1–4^. For example, metasurfaces provide aberration-correction^5^ and diffractive limited resolution^6^ for high-end imaging applications^7^. In addition, polarization-selective focal points^8^, and edge-detections^9,10^ have been demonstrated by spatially engineered polarization profiles of output light from singlet metalenses. Similarly, various unprecedented optical phenomena have been demonstrated with metasurfaces that recode video by exploiting numerous orbital angular momentum (OAM)^11^, focus with near-unity efficiencies at the high-numerical-aperture (NA)^12,13^, and create arbitrary polarization states in three-dimensional (3D) spaces^14^. These sophisticated optical responses are enabled with well-designed artificial structures^15^ and the development of precise nanofabrication methods^16–18^, opening new degrees of freedom for constructing high-end optical devices with compact form factors.

Recently, metasurface research has reached the path to commercialization through the integration of metasurfaces with transitional components such as light-emitting diodes (LEDs)^19^, organic LEDs^20^, vertical-cavity surface-emitting lasers (VCSELs)^21^, charge-coupled devices (CCDs)^22^, microelectromechanical systems (MEMS)^23^, liquid crystals (LCs)^24–26^, waveguides^27^, optical fibers^28^, and even conventional ROEs^29^. By integrating metasurfaces with those optical components, the performance of receiver/emitters has been improved with better receiving/emitting efficiencies. Tunable components offer an effective method for implementing reconfigurable electromagnetic wave manipulation. In addition, wavefronts and dispersion have been precisely manipulated by engineering the optical surfaces of in-couplers, out-couplers, and ROEs. These attempts confirm that metasurfaces can be inserted into current devices via the integration of other standard optical components. Moreover, they indicate several possible methods for constructing structures for practical applications with metasurfaces.

To boost the applications of metasurface-integrated optical systems, a review of integrated metasurfaces is required to pave the way for guiding promising options for high-end optical devices. Although many reviews have carefully organized recent advances in the fundamentals^30–33^, multifunctionality^34–36^, design approach^37–41^, fabrication^42^, and applications^42–47^, reports on the overall concept of integrated metasurfaces for near-future photonic devices are scarce. Certain reviews have focused on emitter-^48–50^, LC-^51,52^, MEMS-^51,52^, waveguide-^53^, and optical fiber-integrated metasurfaces^28^; however, they only focus on the functionality and performances of metasurfaces and do not comment on the practical usage of recent metasurface-integrated optical platforms. From this perspective, this review presents a careful selection of integrated metasurfaces that are expected to be employed in near-future optical devices.

**Fig. 1:**
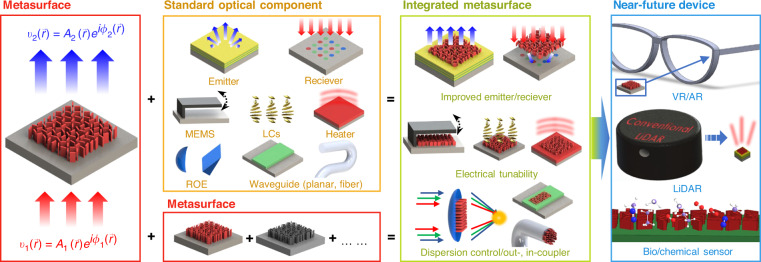
Overall concept of metasurface integration and its promising applications. Recently, metasurfaces have been integrated with standard optical components, such as emitters, receivers, microelectromechanical systems (MEMS), refractive optical elements (ROEs), waveguides, and other metasurfaces. Integrated metasurfaces gain extended functionalities and this will be further applied to a near-future device including virtual reality (VR), augmented reality (AR), light detection and ranging (LiDAR), and bio/chemical sensors

Here, we introduce integrated metasurfaces that are used for hybridization with other standard optical components such as emitters, receivers, MEMS, LCs, heaters, ROEs, planar waveguides, and optical fibers (Fig. 1). In addition, this review covers several metasurface components used in practical photonic systems. In the second half of the review, we discuss the recent development of metasurface-integrated photonic platforms including virtual/augmented reality (VR/AR)^54^, light detection and range (LiDAR), and photonic sensors. In conclusion, we summarize the entire review paper and introduce the main challenge that should be overcome for re-envisioning of near-future optical devices.

## Integration with emitters and receivers

Light emitters and receivers are key components for constructing photonic devices ranging from smartphone cameras to LiDAR. Over the past years, metasurfaces have improved the performance of the emitter and receivers in terms of efficiency and resolution. Enhanced efficiencies reduce the energy consumption of the total optical system, enabling more lightweight with reduced battery sizes. The increased resolution provides an immersive display with high-quality visualization. Furthermore, non-classical light sources such as single-photon and nonlinear emitters have been integrated with metasurfaces, increasing the manipulation capability of a single photon and nonlinear light. Metasurface-integrated non-classical light sources provide on-demand light emitters that have the potential to be used for next-generation optical systems including quantum and neuromorphic computing^55–57^. In addition, metasurface-integrated optoelectronic devices have been recently investigated, enabling flexible photodetectors^58^ and environmental-friendly energy harvesting^59^. Here, this review introduces metasurface-integrated emitters and receivers, along with their performance and potential applications.

### Metasurface-integrated light emitters

Light emitters, also known as light sources, are essential optical components for the construction of display systems. In general, a compact form factor is in high demand with the development of wearable devices; however, conventional systems are still bulky because several ROEs and DOEs are required to create the desired wavefront. Furthermore, the use of several optical components significantly decreases the efficiency of the emitters, which is not compatible with sustainability. Recently, metasurface research has been dedicated to creating desired emission profiles with VCSELs and improving the light extraction efficiencies from LEDs. These efforts provide a valuable possibility for realizing a compact displaying system with a highly immersive image. Also, metasurface-integrated non-classical light sources (e.g., second harmonic generation (SHG), and single-quantum emitters) have been proposed to enhance the capability of light sources. The various metasurface-integrated light emitters are described below.

**Fig. 2:**
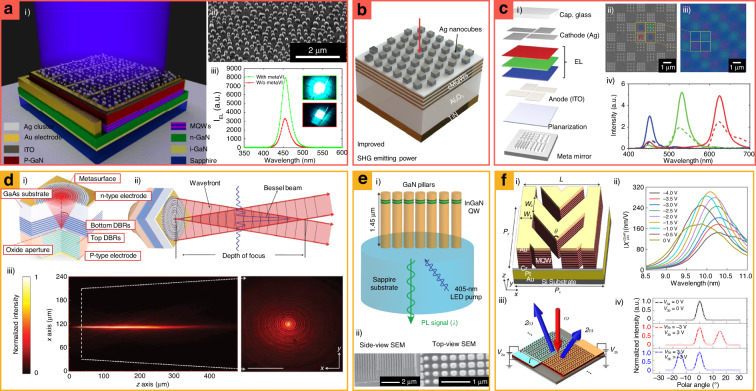
Metasurface-integrated light emitters for (a–c) efficiency improvement and (d–f) wavefront shaping. **a.i** Schematics and **a.ii** an SEM image of disordered Ag nanoparticle-integrated GaN emitters. **a.iii** Electroluminescence (EL) intensity *I*_EL_ with/without Ag nanoparticles, corresponding to green and red curves, respectively. **b** Ag metasurface-integrated second harmonic generation (SHG) emitters. cMQWs: coupled multiple quantum wells. Red arrow: incident direction of a pump laser. **c.i** Schematic of meta mirror-integrated organic light emitting diodes (LEDs). **c.ii** SEM and **c.iii** optical microscope image of the meta mirror. **c.iv** Luminescence intensity comparison of meta mirror-integrated and color-filtered organic LEDs, corresponding to solid and dashed curves, respectively. **d.i** Schematics of metasurface-integrated vertical-cavity surface-emitting lasers (VCSELs), and **d.ii** its Bessel beam generation. DBRs: distributed Bragg reflector mirrors. **d.iii** Measured intensity profiles of the Bessel beam along the propagation direction. **e.i** Schematic of InGaN/GaN quantum-well metasurfaces for unidirectional luminescence. **e.ii** SEM images of fabricated InGaN/GaN quantum-well metasurfaces. **f.i** Meta-atom of MQW-comprised metasurface SHG emitters. **f.ii** Magnitude of *χ*^(2)^ as a function of the pump wavelengths depending on bias voltages. **f.iii** Schematic of metasurface-integrated nonlinear emitters with various artificial structures. **f.iv** Depending on grating patterns and bias voltages, the propagation direction of the emitted light is adjusted. **a** is reproduced with permission from Ref. ^19^ Copyright © 2021, Peng Mao et al., **b** from ref. ^69^ Copyright © 2019 Haoliang Qian, et al., **c** from ref. ^20^. Reprinted with permission from AAAS, **d** from ref. ^21^ Copyright © 2020 Springer Nature, **e** from ref. ^83^ Copyright © 2020 Springer Nature, **f** from ref. ^84^ Copyright © 2020 The Springer Nature

One of the challenges of conventional LEDs is low light extraction efficiencies due to total internal reflection (TIR) at the encapsulation layers, whose critical angle is 30°. To suppress TIR, various nanostructures have been designed to encapsulate layers with random wrinkles^60^ and photonic crystals^61,62^. However, early metasurface designs required a complex fabrication process (e.g., multiple deposition^63,64^, post-annealing^65,66^, and photolithography^67,68^) that incurred high production costs for emitters. More recently, cost-effective index-matching layers with disordered Ag nanoparticles have been proposed for commercial GaN LEDs (Fig. 2a)^19^. The Ag nanoparticles can be manufactured with a single-step fabrication method which is gas-phase cluster beam deposition. The disorder and density of Ag nanoparticles have been optimized by adjusting fabrication parameters, improving light extraction efficiencies by a factor of 1.65 by extracting photons at an incident angle beyond 60°.

The efficiency of SHG emitters has also been improved by combining Ag metasurfaces with TiN/Al_2_O_3_ epitaxial multilayers coupled with multi-quantum wells (MQWs) (Fig. 2b)^69^. The epitaxial multilayers have a TiN/Al_2_O_3_ thickness of 1.0/2.2 nm, producing SHG at 450 nm. The Ag metasurfaces have two plasmonic resonances, which corresponded to fundamental (920 nm) and SHG wavelengths (460 nm). At the resonance wavelength, the Ag metasurfaces generate the desired *z*-direction polarized light, increasing the energy transfer ratio of the incident light onto the coupled MQWs. These Ag metasurface-integrated SHG emitters record conversion efficiencies (10^−4^) higher by several orders of magnitude under an incident pulse intensity of 10 GW cm^−2^ compared to the classical SHG emitters^70^. A similar approach has been continuously investigated with various shapes of artificial materials, such as plasmonic nanocross^71^, T-shaped resonators^72^, split ring resonators^73^, and high-index dielectric gratings^74^. Since metasurfaces can be fabricated on flat surfaces, metasurfaces that improve the efficiencies may be further applied to other types of SHG emitters with 2D materials^48,75^.

Meta mirrors have been implanted in organic LEDs, offering ultrahigh pixel densities (>10,000 pixels) with twice the luminescence efficiency (Fig. 2c)^20^. Commercial organic LEDs comprise two mirrors for the Fabry–Pérot (F–P) cavity and an emissive layer located between the two mirrors. Nanostructures are implanted on a backplane mirror, which is named meta-mirrors, and alter the reflected phases depending on their dimensions^20,76,77^. By optimizing reflected phases, cavities are designed to have individual RGB resonances where luminance efficiencies are the maximum while having the same cavity thickness. Compared to conventional organic LED that has different optical F–P cavity thicknesses depending on its target color, the meta mirrors-integrated organic LED having the physically constant thickness facilitates ultrahigh pixel densities, allowing for a scalable, low-cost nanoimprinting method. With these advantages, it has the potential to be used for commercial VR/AR displays that require ultra-high-resolution pixels with a low production cost.

Wavefront manipulation with illuminants has been achieved by integrating metasurfaces with emitters. Regardless of ever-increasing demands of precise beam shaping from sources, wavefront manipulation of emitted light had not been achieved with conventional ways (e.g., optical waveguides^78^, surface relief structures^79^, etc.). This problem is circumvented by adopting metasurfaces on the VCSELs^80^, collimating light with a divergence angle of 0.83° (Fig. 2d)^21^. Furthermore, metasurface-integrated VCSELs have been used to construct various wavefronts, including those of Bessel beam generation^21^, vectorial holography reconstruction^81^, and beam steering^21,82^. Because both the VCSEL and metasurfaces are compatible with the same CMOS process, they may be easily implanted into a commercial wafer-scale manufacturing process.

Unidirectional luminescence from incoherent LEDs has been demonstrated by implanting InGaN/GaN quantum-well into patterned structures (Fig. 2e)^83^. Unidirectional light emission from LEDs is highly demanded since Lambertian light emission from conventional LEDs induces light losses when applied to paraxial approximated optical systems. Thus, unidirectional luminescence is essential to increase the efficiency of the total optical components, which are designed with commercial ROEs. Unidirectional luminescence has been achieved by InGaN/GaN quantum-well patterns with 100-fold external quantum efficiencies compared to photonic crystal layers. Also, the direction of emitted light can be controlled by varying the radius of patterned cylinders, achieving a steering angle of 80°.

The propagation direction of the nonlinear light has also been controlled using MQW-comprised metasurfaces (Fig. 2f)^84^. Conventionally, heterostructures comprising stacked subwavelength layers can generate nonlinear optical responses with a compact form factor. However, their tunable nonlinear optical responses with high efficiency have rarely been reported. To solve this limitation, MQW-comprised metasurfaces are obtained by engraving patterns on MQWs composed of In_0.53_Ga_0.47_As and Al_0.48_In_0.52_As layers^84^, and implanting two metallic layers applied to bias voltages have been proposed. When the applied voltages are adjusted, the intensity and phase of the emitted SHG light are manipulated at a wavelength of 10 µm, thus providing free-space propagation tuning. This method can be further applied to optical encryptions^85,86^ and nonlinear switching systems^87^ with nonlinear wavefront shaping.

### Metasurface-integrated single-photon emitter

Single-photon control has gained attention in the field of quantum communication, benefiting from its high speed and large information transfer capability compared to those of classical computers. Many computational methods have been developed using various schemes of quantum computation optics^56,88,89^. However, a major challenge encountered is the implementation of computational technologies for physically existing devices. Although photonic machines offering quantum manipulation have been recently proposed^89,90^, characteristics of photons (e.g., spin angular momentum, OAM, optical paths, and frequency) have not yet been fully controlled. Recently, single-photon characteristics have been controlled through the integration of metasurfaces with single-photon emitters, and exotic optical responses have been experimentally achieved using *β-*barium borate (BBO) crystals, quantum-dots, 2D materials, and nitrogen-vacancy (NV) centered diamonds.

**Fig. 3:**
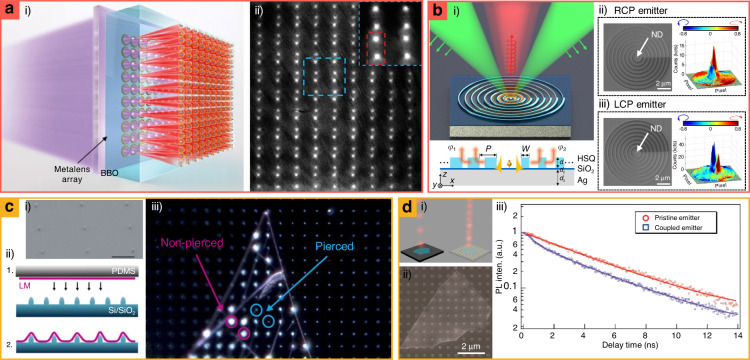
Metasurface-integrated single-photon emitters with (a) *β*-barium borate (BBO) crystals, (b) nitrogen-vacancy (NV) centered diamonds, and (c and d) 2D materials. **a.i** Schematic and **a.ii** optical microscopy image of metasurface-integrated spontaneous photon-emitter. **b.i** Schematics of HSQ metasurface-integrated single-photon emitter. A yellow dot at the bottom schematic presents the NV-centered diamond with **b.ii** right-circularly polarized (RCP) and **b.iii** left-circularly polarized (LCP) light emitters have been demonstrated. The hydrogen silsesquioxane (HSQ) metasurface consists of an azimuthally gradient width. **c.i** SEM image of metasurfaces before 2D emitter integration. **c.ii** Fabrication process of metasurface-integrated 2D emitter. LM: layered materials. PDMS: polydimethylsiloxane. **c.iii** Dark field optical microscopy images of the metasurface-integrated 2D emitters. Blue and red circles: positions of pierced and non-pierced 2D materials, respectively. **d.i** Schematic and **d.ii** SEM images of plasmonic nanocavity integrated 2D emitters. **d.iii** Time-resolved photoluminescence (PL) measurement for comparison of emission rate between conventional (pristine emitter) and plasmonic nanocavity integrated 2D emitters (coupled emitter). **a** is reproduced with permission from ref. ^93^. Reprinted with permission from AAAS, **b** from ref. ^94^ Copyright © 2020 Wiley-VCH, **c** from ref. ^96^ Copyright © 2017 Carmen Palacios-Berraquero et al., **d** from ref. ^103^ Copyright © 2017 American Chemical Society

Emitting multiple entangled photons, also known as multiphoton states-generation, is required to realize quantum computation systems^91^; however, the current spontaneous photon emission is limited by the number of ~20^92^. Through the integration of metalens arrays on BBO crystals, spontaneous photon-emitters have been demonstrated (Fig. 3a)^93^. The metalens arrays focus the incident pump laser (*λ* = 415 nm) on the inside of BBO crystals, triggering a spontaneous parametric down-conversion that converts one high-energy single photon to two lower-energy photons. With metalenses-integrated spontaneous photon emitters, four- and six-photon generation can be achieved, and the emitted photons exhibit indistinguishability from different metalenses. Considering that spontaneous photon emitters have a more compact compactor compared to those of conventional multiphoton emitters, they may be useful for miniaturizing multiphoton-based quantum computing systems.

Metalenses have also been implanted with diamond NV centers, which are promising single photon emitters^94,95^. Consequently, three challenges of conventional emitters are circumvented: (1) limited collection efficiency of photoluminescence (PL), (2) TIR (*θ*_c_ = ~25°) from the host diamonds, and (3) lack of polarization control. Improved PL collection efficiency and suppression of TIR have been achieved through patterning of high-NA metalenses onto diamond surfaces, which collimates the emitted photons from the individual NV center located ~20 µm beneath the surface^94^. Furthermore, polarization splitting with a diamond NV center has been demonstrated with patterned hydrogen silsesquioxane (HSQ) metasurfaces on Ag mirror substrates (Fig. 3b)^95^. The HSQ metasurfaces have HSQ patterns comprising circular nanoridges with azimuthally varied widths, which enable well-defined chirality and high directionality^95^. Using the circular nanoridges, right-, and left-circularly polarized single photons have been experimentally controlled.

Various single-quantum emitters with 2D materials have been integrated with metasurfaces for Purcell enhancements. Deformed 2D materials, which are well-known single photon emitters have been fabricated using metasurfaces as substrates. For example, the precise and accurate positioning of quantum emitters has been demonstrated by depositing 2D materials onto nanopatterned substrates. When 2D materials (e.g., WSe_2_ and WS_2_) are placed on periodically arranged nanopillars, 2D materials emit light where it is distorted but not pierced by nanopatterns (Fig. 3c)^96^. They proved that metasurfaces can be used for scalable quantum emitters by deforming 2D materials, and this method has been further applied to other materials such as single-layered hBN^97^, MoS_2_^98,99^, MoSe_2_^100^, MoTe_2_^101^, and InSe^102^.

Another 2D material, hexagonal boron nitride (hBN) has been integrated with a plasmonic nanocavity array, leading to an enhanced emission rate and reduced fluorescence lifetime (Fig. 3d)^103^. Plasmonic nanocavities support lattice plasmon resonance, which generates a strong localized field around the plasmonic structures. The plasmonic nanocavities exhibit resonance at a wavelength of 641 nm, which is the same as that of the photon sideband of hBN. By coupling plasmonic resonance with the band, plasmonic nanocavity-integrated emitters facilitate lifetime reduction with PL enhancement.

### Metasurface-integrated receiver

Receivers, also known as detectors, are optical components that transfer light energy to electrical signals, thereby enabling the capture and detection of light information^104^. Recently, metasurfaces have been integrated with receivers to increase detection efficiencies, sort input light, and widen field of view (FoV).

**Fig. 4:**
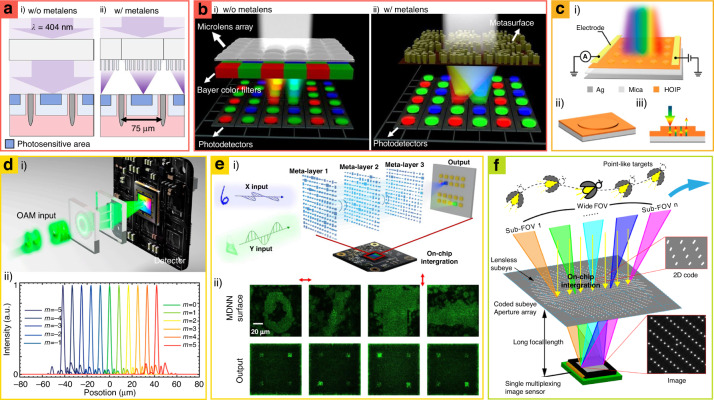
Metasurfaces-integrated receivers for (a and b) efficiency improvement, (c) selective photodetection, (d and e) wavefront sorting, and (f) increasing field of view (FoV). Efficiencies of **a** UV and **b** RGB charge-coupled devices (CCDs) have been improved by integrating metalenses. Compared to **a.i**, and **b.i** CCDs without metalenses, **a.ii** and **b.ii** metalens-integrated CCDs achieve higher efficiencies by focusing the incident light on the photosensitive area. **c.i** Schematics of metasurface-integrated hybrid organic–inorganic perovskites (HOIPs) for optoelectronic devices. Metasurface-integrated HOIPs consist of **c.ii** HOIP meta-atoms and **c.iii** backplane mirrors. **d.i** Schematics of metasurface-integrated CCD for orbital angular momentum (OAM) sorting. **d.ii** Focal point of the metasurfaces depends on the topological charge of OAM. **e.i** Schematics of computation systems with the metasurface-integrated CCD. **e.ii** Depending on the incident wavefront, the focusing points of outgoing light from metasurface-integrated CCDs are varied. **f** Wide FoV with a multiple aperture-integrated CCD. **a** is reproduced with permission from ref. ^22^ Copyright © 2021 American Chemical Society, **b** from ref. ^107^ Copyright © 2022, Xiujuan Zou et al., **c** from ref. ^110^ Copyright © 2022 American Chemical Society, **d** from ref. ^113^ Copyright © 2022 American Chemical Society. **e** from ref. ^116^ Copyright © 2022 Xuhao Luo et al., **f** from ref. ^119^ Copyright © 2022, Li Zhang et al.

The efficiency of CCDs has been improved by integrating metalenses that focus incident light into photosensitive areas^22,105^. In single-wavelength CCDs, metalenses have been used to focus incident unpolarized UV light onto photosensitive areas (Fig. 4a)^22^. When the focal point is located at the center of the photosensitive area, the detection performance of the devices is improved by 9.9%. Similarly, multiwavelength CCDs have been integrated with dispersion-engineered metasurfaces using interleaved GaN^106^, binary-type Si_3_N_4_^107^, and tall Si_3_N_4_ structures^108^. In a specific example, binary-type Si_3_N_4_ achieves efficiencies of 58%, 59%, and 49% at red, green, and blue lights, respectively, which are twice the values of commercial Bayer color filters that filter out wavelength mismatched incident light with a photosensitive area (Fig. 4b)^107^. These metasurface-integrated CCDs innovate information acquisition systems^109^.

Metasurfaces have been integrated with optoelectronic devices to improve photoelectric conversion, enabling efficient energy harvest and information science^110,111^. For example, optoelectronic hybrid organic–inorganic perovskite (HOIP) films have been integrated into metasurfaces, enhancing their optoelectrical conversion in the broadband operating region from ultraviolet to visible (Fig. 4c)^110^. Metasurfaces are directly patterned on HOIP films, and a high refractive index of structured HOIPs provides Mie scattering with strong light confinement. Compared to planar HOIP films, the metasurface-integrated HOIP films exhibit 10 times higher photocurrent at the voltage of 1 V. Similarly, color-sensitive photodetectors have been proposed with silicon–aluminum hybridized metasurfaces^112^. The silicon–aluminum hybridized metasurfaces generate electron–hole pairs with high color selection, achieving submicron photodetectors.

Metasurfaces have given wavefront sorting functionality on CCDs. OAM has been sorted depending on the number of topological charges by metasurface-integrated CCDs^113,114^. Doublet TiO_2_ metasurfaces have been designed for the metasurface-integrated CCDs for OAM sorters, which sort incident OAM light depending on topological charges from −3 to 3 (Fig. 4d)^113^. The first metasurface transfer donut-shaped OAM light to straight lines at Fourier domains. And the second metasurface, located on the Fourier plane, fan-out and focuses the light on CCD detectors. Although the OAM sorting concept had already been demonstrated with spatial light modulators^115^, the doublet TiO_2_ metasurfaces have meaning that they suppress OAM sorting crosstalk by using submicron meta-atom sizes and provide compact OAM sorting devices by reducing the distance between Fourier plane and optical components.

Similarly, complex wavefronts (e.g., hand-written digits, alphabets) have been distinguished by integrating commercial CMOS sensors with serially composited metasurfaces (Fig. 4e)^116^. Polarization-multiplexed metasurfaces have been employed for recognizing complex wavefronts, and optical responses of the metasurfaces have been designed by conventional electronic neural networks. In experiments, triple-layered TiO_2_ metasurfaces are used for recognizing input images, and the output light from the triple-layered TiO_2_ metasurfaces is clustered at the desired spot. Also, metasurface-integrated CMOS sensors distinguished eight different images with high accuracy. This method is potentially applied for computer vision processing and image recognition in automobile cameras.

The CCD detection angle has been steadily improved through the integration of metasurfaces. Compared with single ROE-integrated CCDs, singlet metasurfaces can construct a compact wide-angle detecting optical system, which has single focal distances regardless of the varied incident angles^117,118^. However, early metasurface research suffered from a trade-off between resolution and detection angles. This problem has been circumvented by using metasurfaces composed of multiple apertures whose opening angle is oriented to specific angles (Fig. 4f)^119^. Depending on the incident angle, the multiple apertures vary spot position onto CCDs, and the multiple aperture-integrated CCDs recognize the position of the target by analyzing the spot position. The improved detection angles facilitate various applications in LiDAR and time-of-flight (ToF) cameras^109^.

## Integration with electrically tunable elements

Although metasurfaces have received considerable attention owing to their great attention with potential to replace conventional bulky optics, achieving tunable optical responses of metasurfaces has been challenging due to the static geometries of nanostructures, impeding their versatile applications^35,52,120–122^. To circumvent these limitations, tunable metasurfaces have been extensively studied using various ranges of materials and systems, such as electrical^123–127^, optical^128–130^, and thermal^131^ tuning mechanisms. Among those systems, the electrical tuning mechanism has been actively investigated with metasurfaces since the system enables their fast response time and high feasibility with conventional controllers. Many approaches have been developed to integrate metasurfaces with electrically tunable components including MEMS, LCs, and heaters.

### MEMS-integrated metasurfaces

MEMS provides straightforward geometrical reconfigurability at the micro-scale and has been applied to changing the geometries of metasurfaces. Owing to their high compatibility with mature CMOS technology, MEMS-integrated metasurfaces have garnered significant attention in industries with increasing demands for more multifunctional devices. In this section, we discuss MEMS-integrated metasurfaces including MEMS-actuated metalenses^132–135^, on-chip beam steering devices^23,136–138^, and tunable structural-color pixels^139,140^.

**Fig. 5:**
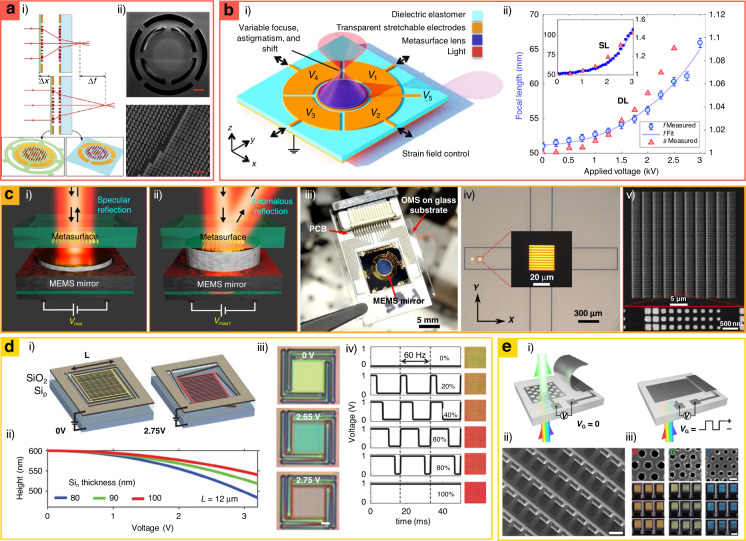
Microelectromechanical systems (MEMS)-integrated metasurfaces for MEMS-actuated (a and b) metalens, (c) beam-steerer and (d and e) structural-color pixel. **a** Tunable dielectric doublet metalens using the MEMS-based SiN_*x*_ membrane. **a.i** Illustration of the operation principle and design. **a.ii** SEM images of the doublet lens system on (top) the metasurface on the membrane and (bottom) its nanostructures (scale bars: 100, 1 μm). **b** Adaptive metalens which can simultaneously control focal length, astigmatism, and shift. **b.i** Device design of the dielectric elastomer metalens comprising the five reconfigurable voltage connections. **b.ii** Experimental focal shifts using the middle electrode V_5_. Solid blue lines, blue circles, and red triangles represent the fit of focal points, focal length, and stretch, respectively, as a function of the applied voltage. **c** Dynamic beam steering device based on the integration of optical metasurfaces and piezoelectric MEMS mirror. **c.i** Operational schemes for 2D wavefront shaping of the device, specular reflection, and **c.ii** anomalous reflection. **c.iii** Image of monolithic integration between MEMS mirror and optical metasurfaces. **c.iv** Optical microscope images and **c.v** SEM images of the 30 × 30 μm^2^ metasurface and 250 nm-period gold meta-atoms in the MEMS-mirror-based dynamic beam steering device. **d** Temporal color mixing device obtained by modulating the gap between two Si layers. **d.i** Dynamic height control of MEMS-integrated tunable temporal color mixing device from 0 to 2.75 V. **d.ii** Mechanical simulation by changing voltages and Si_*n*_ thicknesses (pixel size: 12 μm). **d.iii** Bright-field images of tunable color pixels consist of nanowires. Scale bar: 2 μm. **d.iv** Temporally combined colors by the diverse electric duty cycle. **e** Transmissive adjustable color filters combined with MEMS cantilever. **e.i** Schematic of the design and principle. Transmission can be controlled by a MEMS cantilever. **e.ii** SEM images of MEMS cantilever (scale bar: 50 μm) and **e.iii** SEM and optical microscope images of plasmonic nanohole arrays corresponding transmission colors (scale bars: 250 nm to SEM images and 30 μm to microscope images). **a** is reproduced with permission from ref. ^133^ Copyright © 2018, Ehsan Arbabi et al., **b** from ref. ^134^ Copyright © 2018 The Authors, some rights reserved; exclusive licensee American Association for the Advancement of Science, Distributed under a CC BY-NC 4.0 license http://creativecommons.org/licenses/by-nc/4.0/, Reprinted with permission from AAAS, **c** from ref. ^23^ Copyright © 2021 The Authors, some rights reserved; exclusive licensee American Association for the Advancement of Science, Distributed under a CC BY-NC 4.0 license http://creativecommons.org/licenses/by-nc/4.0/, Reprinted with permission from AAAS, **d** from ref. ^138^ Copyright © 2019 The American Association for the Advancement of Science, **e** from ref. ^140^ Copyright © 2022 The Authors, some rights reserved; exclusive licensee, Distributed under a CC BY-NC 4.0 license http://creativecommons.org/licenses/by-nc/4.0/, Reprinted with permission from AAAS American Association for the Advancement of Science

Early MEMS-integrated metalenses have been developed by exploiting the mechanical deformation of the substrates, varying distances of adjacent metalenses^133^ or nanostructures^141^. For example, MEMS-integrated doublet metasurfaces have been proposed for electrically reconfigurable focal distance (Fig. 5a)^133^. The doublet metalenses are composed of converging and diverging metalenses, and they achieve continuously tunable focal lengths from 635 to 717 µm by changing the distances between the two metalenses at the visible wavelengths. Furthermore, astigmatism and shift have been accomplished with the MEMS-integrated metalens (Fig. 5b)^134^. This MEMS-integrated metalens comprises a centimeter-scale dielectric elastomer metalens with five reconfigurable voltage control electrodes to resolve precise misalignment correction. Similarly, many MEMS-integrated metalenses have been continuously demonstrated with various functions such as a focal length change of 68 μm at the infrared range 1550 nm)^135^ and collection of a beam in the mid-infrared region with a dynamic beam-focusing angle (±9°) which can be used as 2D MEMS scanners^132^.

Dynamic beam-steering metasurfaces have also been demonstrated using MEMS-integrated metasurfaces. For example, MEMS-integrated metal–insulator–metal (MIM) structures have enabled tunable optical responses by mechanically changing the position of the top metallic layers. The resonance wavelength and radiation phase profiles are manipulated, and they form arbitrary wavefront shaping, enabling a dynamic beam deflector^137^. In another example, metasurfaces capable of manipulating complex dynamic 2D wavefronts have been demonstrated through the integration with a MEMS mirror that reduces the distance between metasurface and mirror to generate gap-surface plasmon resonance (Fig. 5c)^23^. This platform enables polarization-independent reflection angle control (0°, 7.7°, and 15.5° in air corresponding to first, second, and third diffraction orders, respectively) with a high operating speed (0.4 ms) and over 50% efficiency. Additionally, MEMS-integrated metasurfaces with Si-air-Si gap-controlled structures have been deployed in dynamic beam steering, resulting in a high tuning speed (>10^5^ Hz) with full phase coverage (0–2*π*)^138^. It also covers the steering angle in the range of 2°–12° regardless of the very low voltage (~3.2 V).

MEMS-integrated color metasurfaces have implemented various tunable structural colorations, achieving low-energy consumption with ultra-high-density resolution and vivid color. MEMS-integrated metasurfaces have been designed by changing the insulator thickness of MIM structures (Fig. 5d)^138^. The MEMS-integrated metasurface has two Si layers, and its distances can be manipulated with low voltage (~2.75 V). By changing the distance, its reflectance spectra are changed, and the dynamic reflective color is exhibited. Most recently, transmitted types of MEMS-integrated color metasurfaces have been designed with an electrically controllable cantilever, which can function as a controller of light that passes through plasmonic nanohole arrays (Fig. 5e)^140^. An ultra-high modulation speed (~800 Hz) with full-color coverage is achieved using this design.

### LC-integrated metasurfaces

LCs have been steadily used for commercial display as electrically tunable waveplates owing to their large refractive index variation in the visible region (Δ*n* = 0.2–0.4)^142^. In the field of metasurfaces, LCs have been recently applied for electrically tunable waveplates to be integrated with polarization-sensitive metasurfaces, and for background index changer of structural materials whose scattering are influenced by the optical index of the host medium. In this chapter, we focus on LC-integrated metasurfaces including tunable structural-color pixels^25,143,144^, spatial light modulators (SLMs)^142,145,146^, and multiplexed metalenses and metaholograms^8,147–150^.

**Fig. 6:**
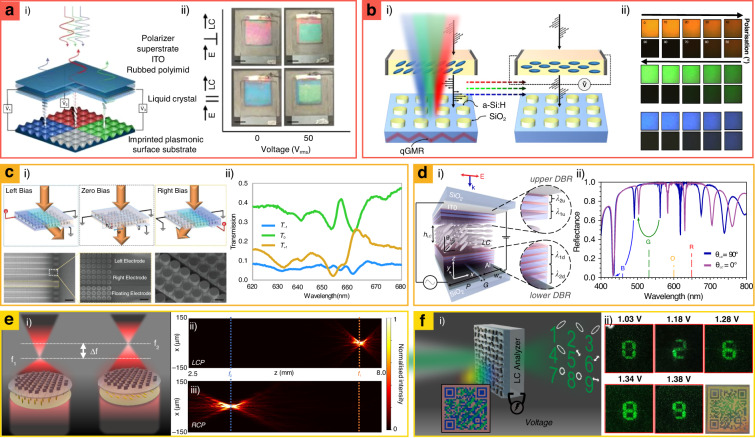
Liquid crystal (LC)-integrated metasurfaces for tunable (a and b) structural color pixel, (c and d) spatial light modulator (SLM), and (e and f) metalens and metahologram. **a** Plasmonic metadisplay composed of addressable structural color using single pixel. **a.i** Schematic of the polarization-dependent full-color pixel design. The ambient white light passes through a polarizer and LC and the plasmonic metasurfaces serve as polarization-dependent absorbers, resulting in different colors reflection. **a.ii** Captured images of the LC-powered plasmonic device depending on incident polarization. Pixel size: 1 × 1 inch^2^. **b** All-dielectric metasurfaces integrated with LC for dynamic colors. **b.i** Schematics of operational principle. **b.ii** Optical microscope images of the full-color gradients by controlling linear polarizer axis from 0° to 90°. Pixel size: 100 × 100 μm^2^. **c** Phase-only SLM device based on LC-integrated metasurfaces. **c.i** Drawings of the design and SEM images of fabricated SLM. Scale bars: 5 μm, 600 nm and 250 nm. **c.ii** Measured transmission at three diffraction orders. **d** LC-coupled SLM based on Fabry–Perot nanocavities. **d.i** Illustrations of the device. **d.ii** Simulated reflectance as a function of wavelength, according to two different LC orientation angles (0° and 90°). **e** Electrically controlled bifocal metalens. **e.i** Schematic of bifocal metalens. The focal lengths are shifted by applying a different electrical voltage. **e.ii** The measured focusing intensity profiles at the *x–z* plane with different incident light, left-circularly polarized (LCP), and **e.iii** right-circularly polarized light (RCP). **f** Bifunctional metasurfaces integrated with LC analyzer. **f.i** Schematic of the device. **f.ii** Different metahologram images were obtained by applying different electrical bias and optical microscope images of a quick response code. **a** is reproduced with permission from ref. ^143^ Copyright © 2017, Daniel Franklin et al., **b** from ref. ^25^ Copyright © 2022, Trevon Badloe et al., **c** from ref. ^142^, Reprinted with permission from AAAS, **d** from ref. ^146^ Copyright © 2022, Shampy Mansha et al., **e** from ref. ^8^ Copyright © 2021 Badloe, T. et al. Advanced Science published by Wiley‐VCH GmbH, **f** from ref. ^150^ Copyright © 2021, Inki Kim, et al.

Tunable color pixels have been demonstrated with two types of metasurfaces: (1) LC-integrated plasmonic metasurfaces, and (2) LC-integrated dielectric metasurfaces. The former type has been designed in both reflectance^143^ and transmissive variations^144^. Reflective-typed LC-integrated plasmonic metasurfaces have covered RGB coloration with voltage regulation, and their reflectance varied according to the arrangement of electric field-sensitive LCs (Fig. 6a)^143^. In the case of transmissive types, polarization-sensitive color metasurfaces have been implanted with LCs, and they have demonstrated dynamic reflectance with low voltage (<5 V)^144^. The polarization-sensitive color metasurfaces have been applied for tunable color tags. On the other hand, all-dielectric metasurfaces can provide directional Mie-scattering without Ohmic losses at the visible spectrum, enabling vivid structural coloration with subwavelength pixels. With this advantage, dielectric metasurfaces have been integrated with electrically tunable LCs, providing gradient colors for photorealistic applications **(**Fig. 6b)^25^. These devices fully control the on–off states of each pixel and thus they cover the full RGB color gamut including white and dark black without a second polarizer by mixing the reflectance spectra, which has not been realized in the previous tunable structural color pixels. This approach achieves ultra-high-resolution tunable color printings and multicolor cryptographies.

LC-integrated metasurfaces have accomplished abrupt phase modulation via the application of spatially different biases on the LC, overcoming limitations on the pixel sizes of conventional spatial light modulators (Fig. 6c)^142^. One-dimensional transmissive metasurface-based SLMs have been demonstrated, and they achieve a transmittance efficiency of 36% and an FoV of 22° with a small pixel size (~1 μm). However, they operate at a single monochromatic wavelength. To overcome this limitation, LC-coupled SLMs based on Fabry–Perot nanocavities have been realized to operate at RGB wavelengths (Fig. 6d)^146^. They are composed of sub-micrometer LC cells, leading to a drastic improvement in the response time and interaction between adjacent pixels. These designs can be further expanded to metasurface-based 2D SLMs, thereby rendering the construction of an ultra-high resolution, large viewing angle device possible.

LC-integrated metalenses^8,147,148^ and metaholograms^149–152^ have been extensively studied to realize tunable focal spots and multiplexed images, respectively. In the case of metalenses, tunable bifocal metalenses have been designed using LCs that act as electrically tunable waveplates (Fig. 6e)^8^. The tunable bifocal metalenses experimentally demonstrate two variable focal lengths of 7.5 and 3.7 mm with 44% focusing efficiency. Similar results have been reported with graphene electrodes, wherein a broadband achromatic performance in the range of 0.9–1.4 THz is measured^147^. In the case of a metahologram, LC-integrated bifunctional metasurfaces that project color prints in white ambient light and exhibit holography under coherent laser irradiation have been demonstrated (Fig. 6f)^150^. This configuration is used as a photonic security platform through its encryption into a QR code with a color print and the encryption of numbers as polarization-sensitive vectorial holography^150^.

### Heater-integrated metasurfaces

Heater-integrated metasurfaces exploit the thermo-optic effect, which describes the change in the refractive index in response to temperature, to change their optical responses. Phase change materials (PCMs), such as vanadium dioxide (VO_2_) and chalcogenide alloys, are typically used owing to their large variable refractive index range. When designing meta-atoms with these PCMs, the optical responses of metasurfaces are manipulated by changing temperature using electrical heaters. This chapter introduces the achievements of electro-thermal modulation of PCMs, heater-integrated VO_2_^153–156^, and chalcogenide metasurfaces^157–159^.

**Fig. 7:**
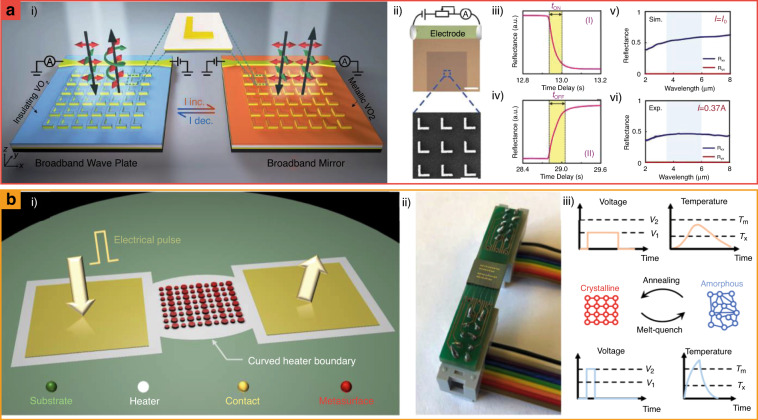
Heater-integrated metasurfaces that consist of (a) VO_2_ and (b) Ge_2_Sb_2_Se_5_Te (GSST). **a** Electrically tunable VO_2_ metasurfaces for dynamic phase modulation. **a.i** Schematics of heater-integrated VO_2_ metasurfaces. **a.ii** Heat is induced by electric voltage. The measurement of **a.iii** heating and **a.iv** cooling time. **a.v** and **a.vi** The optical responses are varied according to the applied electric current on electrodes. **b** Electrically reconfigurable GSST metasurfaces with a microscale heater. **b.i** Schematics and **b.ii** the photo of heater-integrated tunable metasurfaces. **b.iii** Depending on heating time and temperature, the phase of GSST is varied. *T*_m_: melting point, *T*_x_: crystallization temperature of GSST. **a** is reproduced with permission from ref. ^156^ Copyright © 2021 Wiley-VCH, **b** from ref. ^158^ Copyright © 2021 Springer Nature

Heater-integrated VO_2_ metasurfaces have been designed by exploiting the metal-insulator phase transition between 300 and 340 K^160^, providing various tunable optical responses including broadband tunable resonators^154^, switchable waveplates^153,156^, and phase modulators^155^. Specifically, these varied optical responses are defined by the geometries of the VO_2_ structures, and their temperatures can be controlled using electrical heaters. For example, when designed as “L” shaped structures, heater-integrated VO_2_ metasurfaces exhibit tunable functionality between half- and quarter-wave plates (Fig. 7a)^156^. The modulation speeds of the heater-integrated VO_2_ metasurfaces approach 65 and 245 ms when heating and cooling, respectively. Furthermore, the modulation speeds can be varied depending on the geometries of the VO_2_ metasurfaces.

Chalcogenides are attracting significant attention as next-generation application materials owing to their non-volatile characteristics. Ge_2_Sb_2_Se_5_Te (GSST) has been applied to heater-integrated tunable metasurfaces (Fig. 7b)^158^. It comprises nanostructured GSST on microheaters and achieves reversible tunability. Consequently, beam-steering and tunable reflectance have been experimentally demonstrated in the infrared region. Additionally, various PCMs (e.g., Sb_2_S_3_^157^, and Sb_2_Se_3_^157^) metasurfaces have been actively exploited to realize programmable or tunable optical components with various types of heaters such as indium titanium oxide (ITO) in the visible^157^. Owing to the development of various PCMs for photonic platforms, heater-integrated metasurfaces are a promising option for practical optical platforms.

## Integration with conventional optical elements

As the demand for high-end LiDAR and VR/AR technologies has increased, optical systems have been constructed using a series of optical components, including lenses, waveguides, and optical fibers. Metasurfaces offer improved functionality while reducing the size and weight of these optical devices for LiDAR and VR/AR applications. Furthermore, metasurfaces have expanded their application field to include optical communication and computing, which has gained attention due to its bright prospects for high integration density and reduced heat generation. This review introduces metasurface-integrated refractive optical elements and waveguides, along with their prospective applications.

### Metasurface-integrated refractive optical elements

From telescopes to microscopes, ROEs are essential elements for various instruments and have aided in significant scientific discoveries and applications. Conventional optical elements (e.g., prisms and lenses) manipulate light paths by employing refraction to obtain the desired optical functionality for practical photonic devices. However, conventional optical components are unable to fully control the optical dispersion, resulting in spherical aberration, surface distortion of an image, and chromatic aberration. These are attributed to the limitation of the fabrication process of ROEs, which cannot construct an ideal curve of the interfaces. These limitations are solved by attaching metasurfaces on interfaces of ROEs, realizing unprecedented wavefront manipulation with spatially engineered dispersion.

**Fig. 8:**
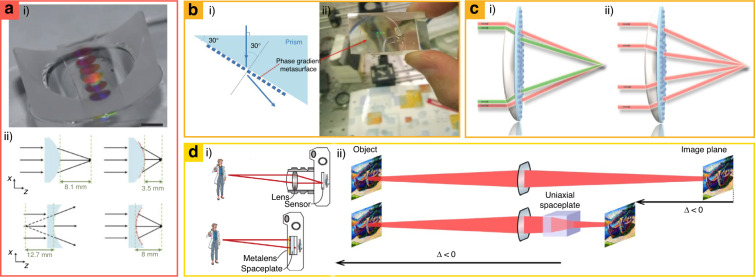
Metasurfaces-integrated refractive optical elements for (a) modifying optical function from geometry, (b and c) dispersion control, and (d) miniaturizing. **a.i** Metasurfaces-attached concave glass cylinder behaving like converging aspherical lens. **a.ii** Focal distance of 8.11 mm of a converging cylindrical lens is modified to 3.5 mm. Concave glass cylinders with a focal distance of -12.7 mm behave as an aspherical lens having a focal distance of 8 mm. **b.i** Schematic of the achromatic system. **b.ii** Experimentally realized achromatic optical component. Schematic of the **c.i** chromatic aberration corrected lens and **c.ii** spherical aberration corrected lens. **d.i** Standard camera (top) and spaceplate added camera (bottom). **d.ii** The spaceplate advances the image’s focal plane by -3.4 mm. **a** is reproduced with permission from ref. ^161^ Copyright © 2016, Seyedeh Mahsa Kamali et al., **b** from ref. ^162^ Copyright © 2019 Wiley-VCH, **c** from ref. ^29^ Copyright © 2021 Optica Publishing Group, **d** from ref. ^164^ Copyright © 2021 Orad Reshef et al.

Metasurfaces have been integrated with conventional cylindrical and spherical refractive lenses to decouple the optical function from the ROE geometries (Fig. 8a)^161^. Traditionally, rays of light are defined by Snell’s law at the interfaces of ROEs; therefore, the function of ROEs is constrained by their physical geometries. Recently, conformal flexible dielectric metasurfaces have been used to convert cylindrical lenses into aspherical lenses for decoupling from physical geometries. The metasurfaces consisted of Al_2_O_3_-capped amorphous Si nanoposts embedded in a PDMS film. With the flexibility of PDMS film, the metasurfaces can be attached to arbitrarily shaped ROEs such as concave and convex reflecting lenses. The focal spot is adjusted as well, for example, 8.1 to 3.5 mm for a converging cylindrical lens (radius: 4.13 mm), and -12.7 to 8 mm for a concave glass cylinder (radius: 6.48 mm). Consequently, metasurfaces have contributed to decoupling geometries of ROEs by manipulating phase profiles of their surfaces, allowing for distortion correction of existing optical components even with a small volume and weight gain.

Aberrations that are typically handled by cascading several lenses are among the most important issues in refractive optics. Metasurfaces can also be utilized to control optical dispersion without stacking multiple optical components. For example, the aberration of prisms (Fig. 8b)^162^ and refractive lenses (Fig. 8c)^29^ have been corrected using metasurfaces. When metasurfaces are attached to the surfaces of ROEs, the desired phase profile is analytically derived^163^ considering chromatic and spherical aberration. Consequently, 80% of chromatic and 70% of spherical aberration are compensated^29^. Thus, artificially engineered interfaces with metasurfaces may provide compact imaging devices through the replacement of multiple systems of ROEs.

Miniaturization has been attempted by replacing conventional ROEs with metasurfaces (Fig. 8d)^164^. Using metasurfaces, spaceplates with an effective thickness *d*_eff_ larger than the physical thickness *d* have been designed. Spaceplates with *d*_eff_ > *d* are realized based on the equation^165^ *φ*_SP_(*k*_*x*_, *k*_*y*_, *d*_eff_) = *d*_eff_(*|***k***|*^2^−*k*_*x*_^2^−*k*_*y*_^2^)^1/2^, where **k** is the momentum vector, *k*_*x*_ and *k*_*y*_ are the propagation vectors of the *k* momentum vector, and *φ*_SP_ is the imparted phase from the spaceplate. Two types of spaceplates are designed: (1) alternating layers of subwavelength silicon and silica to induce collective optical responses, named nonlocal metamaterials, and (2) a uniaxial birefringent medium with an ordinary refractive index larger than the extraordinary one. The metamaterial spaceplate exhibited a compression factor *R* of ~5 and a polarization-independent response. In contrast, the uniaxial spaceplate shows a small compression factor of *R* = 1.12; however, it is found to be broadband in the visible, achromatic, and high NA with a high transmission efficiency. Because the reduction of air gaps between optical lenses gaining traction in the current consumer device market to miniaturize the total optical imaging systems^166^, this result shows that the metasurface can be a promising optical component in future imaging systems with a compact form factor.

### Metasurface-integrated planar waveguides

Compared to electrical wires that are used for electrical computers, electromagnetic wave guiding offers the advantage of low heat generation and solves the integration density problem, enabling high-speed optic computing^167^. Thus, several applications have been reported, enabling high amounts and rates of data transmission with low power consumption^168^. Optical components for electromagnetic wave guiding further extended their ability through their integration with metasurfaces owing to the development of nanofabrication. This has resulted in the fabrication of various sophisticated photonic chips such as photonic-integrated circuits (PICs), waveguides, and metaphotonics^169,170^.

**Fig. 9:**
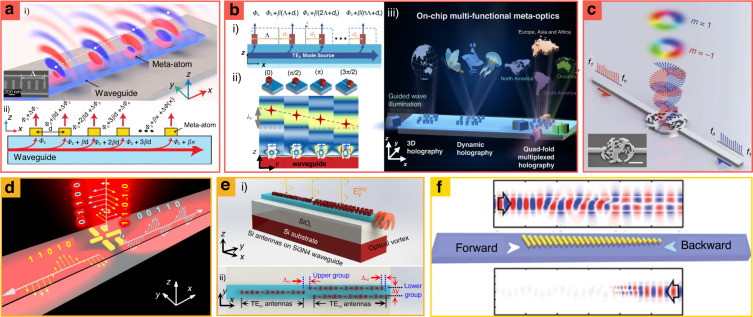
Metasurfaces-integrated planar waveguides for (a–c) structured light, (d and e) demultiplexing, (f) diode. **a.i** Schematic of a guided wave-driven metasurface and **a.ii** wavefront formation of the extracted wave. **b.i** Schematic of a wavefront formation with imparting. **b.ii** Simulated electric field distribution (*E*_*y*_) for the phase shift of 0, *π*/2, *π*, and 3*π*/2. **b.iii** Schematic illustrating novel on-chip meta-holography applications, including multiplane 3D holography, dynamic holography, and quad-fold multiplexed holography. **c** Schematic of the broadband multiplexed OAM emitter. **d** Waveguide-integrated plasmonic nanoantenna that enables mode-selective polarization (de)multiplexing. **e.i** Schematic of the chip-integrated orbital angular momentum generator and **e.ii** its top view. **f** Gradient metasurface on the waveguide inducing asymmetric transmission. **a** is reproduced with permission from ref. ^27^ Copyright © 2020 The Authors, some rights reserved; exclusive licensee American Association for the Advancement of Science, Distributed under a CC BY-NC 4.0 license http://creativecommons.org/licenses/by-nc/4.0/, Reprinted with permission from AAAS, **b** from ref. ^173^ Copyright © 2022 Wiley-VCH, **c** from ref. ^174^ Copyright © 2018, Zhenwei Xie et al., **d** from ref. ^175^ Copyright © 2017 The Authors, some rights reserved; exclusive licensee American Association for the Advancement of Science, Distributed under a CC BY-NC 4.0 license http://creativecommons.org/licenses/by-nc/4.0/, Reprinted with permission from AAAS, **e** from ref. ^176^ Copyright © 2020 Optica Publishing Group, **f** from ref. ^178^ Copyright © 2019 American Chemical Society

One example of using a meta-structure with a PIC is coupling guided waves to free space. When using a PIC for free-space wavefront manipulation, the conventional method involves the use of edge couplers^171^ and surface gratings^172^; however, they cannot cover full-2*π* phase to construct flexible wavefront control. In contrast, the arrays of a grating are more versatile, although they require considerable space, and high-order diffractions generate a loss. However, by placing subwavelength-sized Au/SiO_2_/Au with a metal-dielectric-metal sandwiched configuration on top of the waveguide, each meta-atom can extract and mold guided waves into the desired free-space optical modes (Fig. 9a)^27^. The phase of the extracted wave is calculated as *∅*_0_ + *β*_*x*_ + ∆*∅*(*x*), where *∅*_0_ and ∆*∅*(*x*) are the initial phase of the incidence and the abrupt phase change by metamaterials, respectively; *β*_*x*_ is the phase accumulation from the propagation of the guided wave. This type of structure can enable tightly squeezed optical components by reducing the sizes of light sources.

Using the wavefront manipulation ability of metasurface-integrated out-couplers, various multiplexing holograms have been demonstrated with on-chip 3D sliced holography (Fig. 9b)^173^. Three different letter images are reconstructed at different vertical distances from the metasurface chip. The desired phase profiles of the outgoing wavefront are encoded by changing the position of the α-Si meta-atom, which is placed on top of the Si_3_N_4_ waveguide. The phase of the extracted wave is *∅*_0_ + *β*(*nΛ* + *d*_*n*_), where *∅*_0_ is the initial phase, *β* is the propagation constant, *Λ* is the array period (360 nm), and *d*_*n*_ is the displacement of meta-atoms. It has also been used to construct quad-fold multiplexed images depending on the input direction of light originating from different waveguides.

The creation of OAM light has been demonstrated using an out-coupler of the meta-grating (Fig. 9c)^174^. The out-coupler had two waveguide arms. The *m*_1_ order OAM mode is produced by light from the left arm, and the *m*_2_ order is produced by light from the right arm under broadband input signals (1450–1650 nm). The meta-grating is designed using a global optimization process, which combined annealing and a genetic algorithm to calculate the refractive index distribution. The proposed OAM emitters have also been used for a commercial frequency-division multiplexing system for new high-capacity communication applications.

The integrated nanoantenna is used as a mode demultiplexer with high-bit rate signal transmission (Fig. 9d)^175^. The gold nanoantenna is placed on the silicon waveguide, which can couple *x*-polarized incident light from free space to the TM mode, and the other can couple *y*-polarized incident light to the TE mode. By coupling different light polarizations vertically to different individual waveguide modes, the segregation of optical signals with distinct polarizations has been demonstrated through the separation of the direction of light traveling. This method can be further applied to integrated quantum optics, whose polarization control is a crucial degree of freedom for producing entanglement.

Metasurface-integrated planar waveguides have achieved a coupling efficiency higher than those of the previous works^175^ by 10 times (67%) by rigorously applying phase matching conditions (Fig. 9e)^176^. The phase-matching condition is derived from the Jones matrix model and generalized Snell’s law. The nanostructures comprise Si nanoantennas on Si_3_N_4_ optical waveguide in the vicinity of a telecommunication wavelength of 1.55 μm. The chip-integrated twisted light generator is also described to show the mode-control flexibility, which coupled free-space linear polarization into 1*ħ* OAM.

Although most previous studies on metasurface-integrated waveguides have focused on controlling the characteristics of light when it is going into or out of waveguides, nanoantennas can control guided waves^177^. For example, on-chip asymmetric propagation by phase-gradient metasurface has been accomplished over a broadband THz spectrum (Fig. 9f)^178^. Depending on the propagation direction and polarization, it decreases or guides light through waveguides. The principle behind this is based on the asymmetrically imparted momentum at interfaces with phase discontinuities as represented in *k*_*x*_^out^ = *k*_*x*_^in^–*N*Δ*Φ*/*Λ*_*x*_, where Δ*Φ* and *Λ*_*x*_ are phase difference and periods, respectively. This method can facilitate the development of THz-integrated functional devices. In addition, various waveguided-integrated metasurfaces such as spatiotemporally modulated metasurface^179^, superheterodyne metasurface^180^, and metasurface-assisted second harmonic generation^181^ have been steadily proposed.

### Metasurface-integrated optical fibers

Since optical fibers can guide electromagnetic waves with highly flexible forms, they have been widely used in various fields of photonic devices. However, the commercial optical fiber components are exceedingly large, preventing them from being compact in-fiber optical systems. To solve this problem, metasurfaces have been actively investigated for long-haul operation with effective light directing with low optical loss.

**Fig. 10:**
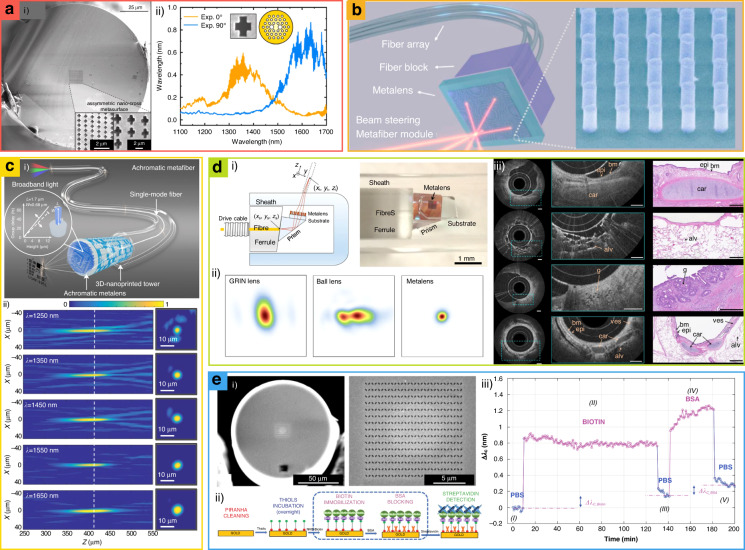
Metasurface-integrated fibers for (a) filtering, (b) steering, (c) focusing, (d) imaging, and (e) sensing. **a.i** SEM images of nanopatterned fiber for polarization-sensitive optical response. **a.ii** Polarization-sensitive transmittance spectra of the fiber. **b** Metasurface-integrated fiber for beam-steering. **c.i** Schematic of the achromatic metalens-integrated fiber for focusing telecommunication range. **c.ii** Experimentally measured point-spread functions of the metafiber. Left: longitudinal planes. Right: transverse planes at five different wavelengths. **d.i** Schematic (left) and photographic image (right) of the metasurface-integrated fiber for tomography. **d.ii** Measured focal spot profiles using a graded index (GRIN) optical coherence tomography (OCT) catheter, a ball lens catheter, and the nano-optic endoscope at 1310 nm wavelength. Nano-optic endoscope shows smaller astigmatism. **d.iii** Ex vivo human lung resections (first, second, and third line) and in vivo in the upper airways of a sheep (fourth line) endoscopic imaging using the nano-optic endoscope. Structural features are clearly visible. **e.i** SEM images of fabricated metasurface-integrated fiber for chemical sensing. **e.ii** Illustrative drawing of the biological protocol. **e.iii** Real-time wavelength shift of the phase-gradient optical fiber meta-tip. **a** is reproduced with permission from ref. ^182^, © 2022 Indra Ghimire et al., published by De Gruyter, Berlin/Boston, **b** from ref. ^183^ Copyright © 2022 American Chemical Society, **c** from ref. ^184^ Copyright © 2022, Haoran Ren et al., **d** from ref. ^185^ Copyright © 2018 Springer Nature, **e** from ref. ^189^ Copyright © 2020 Wiley-VCH

In-fiber polarization-dependent optical filters have been demonstrated by asymmetric nanostructure patterning a plasmonic metasurface on polarization-maintaining photonic-crystal fibers (PM-PCFs) and conventional single-mode fibers (Fig. 10a)^182^. Polarization-dependent transmission with an efficiency of up to 70% in the telecommunication wavelength has been experimentally demonstrated. This result shows that the metasurface filter can be implemented in standard optical fiber, enabling a wavelength-selective filter. This filter can be used for systems that require precise polarization control even in long distances or the existence of external perturbations such as bending of fiber or mechanical vibration.

Additionally, beam-steering metafiber modules have been proposed through the integration of metalens with fiber arrays (Fig. 10b)^183^. To construct a beam-steering metafiber module, metalenses are first fabricated on a SiO_2_ substrate and then attached to the end of the single-mode fiber arrays. The outgoing light from different fibers is steered in different directions according to the phase profiles of the quadratic metalenses, achieving a large FoV of up to 60° at *λ* = 1.55 µm. Furthermore, a LiDAR application for parking-space monitoring using the proposed 2D beam steering metafiber module working in a scanning mode has been demonstrated. Moreover, by enlarging the fiber array and expanding the metalens, a larger scale with improved scanning precision can be achieved.

3D achromatic metalenses have been attached to the end-facet of a commonly used single-mode fiber (SMF-28) has been demonstrated (Fig. 10c)^184^. Conventional fibers suffer from dispersion when light is guided, and it is strongly important because fiber is mostly used for long-distance communication. The designed metafiber is polarization insensitive considering perturbation in the fiber and achromatic in 1.25–1.65 µm range of near-infrared telecommunication wavelengths, covering the entire single-mode domain of the fiber used in commerce. The degree of freedom is increased by one dimension by varying the height of the nanopillar and it is fabricated by 3D laser nanoimprinting via two-photon polymerization using a femtosecond laser. The upper bound *κ* of the time-bandwidth product *κ* ≥ Δ*T*Δ*ω* in an achromatic metalens is significantly increased by the height degrees of freedom unlocked in a 3D nanopillar meta-atom (up to 21.34). This resulted in a broad group delay modulation range from −8 to 14 fs. As a proof-of-concept, by using this thin and flexible achromatic metalens-attached fiber for fiber-optic confocal imaging, clear and in-focus images under broad-band light illumination have been provided.

A metasurface integrated with fiber can be applied to endoscopic optical bioimaging (Fig. 10d)^185^. Specifically, metasurfaces integrated with fibers are used as optical coherence tomography (OCT) catheters. Standard commercial catheters such as the graded-index (GRIN) lens-prism configuration^186^ or angle-polished ball lens^187^ have asymmetrical curvatures in the transverse plane of the cylindrical outer protective sheath, which causes aberrations such as astigmatism. Tangential and sagittal resolutions are influenced by depth; the smallest measured FWHMs are 6.37 (tangential) and 6.53 μm (sagittal). The application of a metasurface achieves near-diffraction-limited imaging by nullifying non-chromatic aberration and reducing the tradeoff between the depth of focus and transverse resolution. Considering that bioimaging with metasurfaces such as high-resolution tomography^188^ has been continuously developed, fiber-integrated metasurfaces will be a promising option for a practical bio-imaging platform.

In addition to bioimaging, a lab-on-fiber biosensor can be realized by integrated metasurfaces. Biosensing with a phase gradient plasmonic metasurface has been demonstrated to capture a wavelength shift by analyzing local variations of the refractive index when the sensing material is attached. The integrated metasurface results in a unique biosensing platform with extremely high sensitivity for the detection of biomolecular interactions of streptavidin (Fig. 10e)^189^. The phase gradient increases the coupling of the incident field to the plasmonic resonance, which allows a larger field enhancement to improve sensitivity with a low fabrication cost. Additionally, owing to its intrinsic compatibility with medical catheters and needles, liquid biopsy applications involving real-time diagnosis in various body regions are possible.

## Optical platforms with composed metasurfaces

Metasurfaces comprising two or more metasurfaces can realize multi-functionality with various degrees of freedom, extending the functionality of singlet metasurfaces. Singlet metasurfaces suffer from many trade-offs when multi-functionality is encoded. For example, when multiple metaholograms and multiple focal lengths are encoded in singlet metasurfaces, the efficiency of each mode is significantly decreased. Further, in dispersion control, performance aspects of metalenses such as the diameter, efficiency, and NA are sacrificed to acquire the exact group-delay^190–192^. In this context, composed metasurfaces have been investigated to solve these problems, and variously composed metasurfaces have been customized by respectively changing the light properties of each surface. With a composed structure, various wavelength and polarization multiplexing processes have been demonstrated for practical spectroscopy^193^ and polarimetry^194^ application. In this chapter, this review introduces additional functionalities of composed metasurface in terms of multiplexing, dispersion control, and tunability.

### Composed metasurfaces

**Fig. 11:**
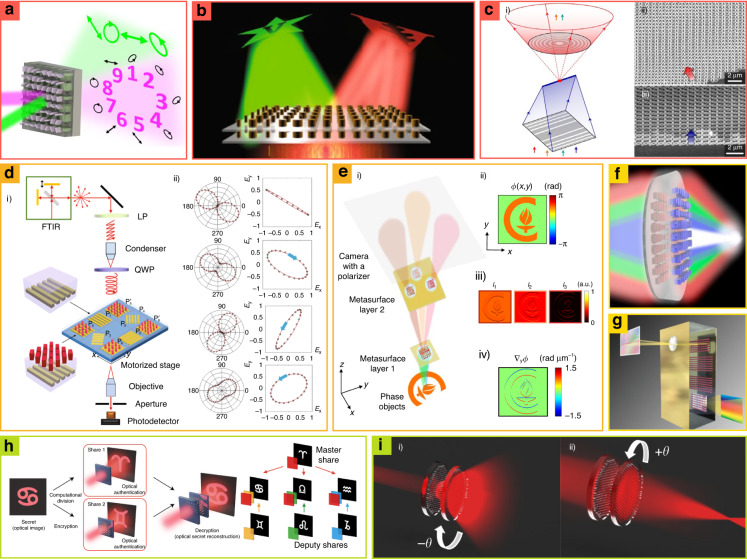
Optical platform of composed metasurfaces for (a-c) wavelength decoupling, (d and e) polarization decoupling, (f and g) dispersion control, and (h and i) tunability. **a** Photonic encryption platform that is composed of ultraviolet and visible metasurfaces. **b** Bilayer metasurfaces independently control two infrared frequencies. **c** Composed non-local metasurfaces. **c.i** Schematics of composed non-local metalenses operating with different wavelengths. SEM image of **c.ii** diverging and **c.iii** converging non-local radial metalenses, respectively. **d** Full-Stokes polarimetric measurement setup using composed metasurfaces. **d.i** Ommatidium-like double-layer metasurface (ODLM) for circular polarization filters with the motorized stage. **d.ii** Comparison of detected polarization using metasurfaces with conventional analysis. **e** Quantitative phase gradient microscope (QPGM) with metasurfaces. **e.i** Schematics of the optical setup for QPGM. **e.ii** Image of the target object. **e.iii** Three differential interference contrast (DIC) images were obtained with multiple birefringent metasurfaces. **e.iv** Phase gradient images formed from DIC. **f** Polarization-independent doublet metalens for collecting chromatic aberration. **g** Hyperspectral imager (HSI) composed of four metasurfaces. **h** Tunable holography encryption system via cascaded metasurfaces. Metasurfaces are classified as master shareholder and deputy shareholder. Master shareholders can be combined with other deputy shareholders showing a different secret image along the combination. **i** Composed Moire metalenses for tunable focal length. **i.i** Negative rotation angles make negative tunable focal lengths. **i.ii** Positive rotation angles make positive tunable focal length. **a** is reproduced with permission from ref. ^195^ Copyright © 2022 American Chemical Society, **b** from ref. ^196^ Copyright © 2019, You Zhou et al., **c** from ref. ^199^ Copyright © 2022, Stephanie C. Malek et al., **d** from ref. ^194^ Copyright © 2019, Ali Basiri et al., **e** from ref. ^200^ Copyright © 2019 Springer Nature, **f** from ref. ^202^ Copyright © 2022 American Chemical Society, **g** from ref. ^193^ Copyright © 2019 American Chemical Society, **h** from ref. ^206^ Copyright © 2021 The Authors, some rights reserved; exclusive licensee American Association for the Advancement of Science, Distributed under a CC BY-NC 4.0 license http://creativecommons.org/licenses/by-nc/4.0/, Reprinted with permission from AAAS, **i** from ref. ^208^ Copyright © 2020 Optica Publishing Group

Stacking the metasurfaces enables various multiplexing functionalities (e.g., varied focal length, and multiple metaholography), while they prevent the undesired diffraction and low resolution caused by interleaved design with large periods. Composed metasurfaces have been demonstrated for multi-wavelength holography, polarization-independent holography, and multifunctional nonlocal metasurfaces. Composed metasurfaces for wavelength-multiplexed holography show two independent holographic images under laser light of ultra-violet (UV) and visible wavelengths (Fig. 11a)^195^.

The first layer of metasurfaces has a manipulation efficiency of 18% in the visible range, although it cannot manipulate UV (manipulation efficiency of 0%). However, the second layer has high efficiency (72%) at *λ* = 325 nm with lower efficiency (3.4%) at *λ* = 532 nm. By stacking the two metasurfaces, UV and visible holography can be decoupled and utilized as optical encryption platforms. Another example is polarization-independent holography, which has been demonstrated by composing two metasurfaces^196^. Because composed metasurfaces can control two infrared wavelengths (1180 and 1680 nm) without efficiency degradation of singlet interleaved metasurfaces owing to space-filling limitations and cross-talk^197,198^, they have a wide phase map with high transmittance at two wavelengths. Consequently, two independent hologram images at two wavelengths can be produced with high efficiency of 48.1% and 50.3% at 1180 and 1680 nm, respectively (Fig. 11b)^196^. In contrast to the metasurfaces introduced above, non-local metasurfaces control both spatial and spectral lights^199^, breaking bound states in the continuum (BICs), where light interacts very strongly with the material and is confined by an infinite *Q*-factor. The non-local metasurfaces have been designed with symmetry-broken meta-atoms to create quasi-BIC (q-BIC) that allow for a leaky state, where confined light merges with phase delay at a specific wavelength. Using this, composed non-local metasurfaces manipulate the wavefront only at multiple resonant wavelengths and allow light transmission without modulation at non-resonant wavelengths, where multiple layers comprise independent meta-atom cells in the q-BIC mode (Fig. 11c). Non-local metasurfaces also make the stacking process easy with the selective response at a specific frequency, preventing it from degraded functionality from misalignment contrary.

As composed metasurfaces can fully control the polarization and phase of light without high optical losses, they have been applied for highly efficient full-Stokes polarimetric^194^ and phase grating measurements^200^. Full-Stokes polarimetric measurements have been proposed with an ommatidium-like double-layer metasurface (ODLM) design, where each metasurface acts as a quarter-wave plate (QWP) and a linear polarizer (LP) (Fig. 11d). Full-Stokes polarimetric detection is realized on one chip via integration of two ODLMs with nanowire gratings in four different orientations. Random incident light is distributed with six polarization filters and the Stokes parameter is extracted by a photodetector (in Fig. 11d, Additional two filters are for another wavelength)^194^.

A miniaturized quantitative phase gradient microscope (QPGM) has been demonstrated with multilayer birefringent metasurfaces, where a phase gradient image is produced from quantitative phase data (Fig. 11e)^200^. Mimicking a birefringent material that separates the polarization states (TE and TM), the first-layer birefringent metasurface produces two images along the TM and TE modes and separates these images equally into three directions of the second layer composed of three birefringent metasurfaces. They have received split images from the first birefringent metasurfaces to form three different differential interference contrast (DIC) images via the utilization of different phase offsets of the received TM and TE images. Consequently, the QPGM obtains three DIC images simultaneously and produces a phase-gradient image by combining these images.

Multiple layers facilitate dispersion control with low design complexity compared to singlet achromatic metalenses that require various meta-atom designs^201^. Recently, bilayer achromatic metalenses have been achieved with a simple meta-atom design with a high NA (0.8) and large diameter (1 mm) (Fig. 11f)^202^. The metalenses comprise cylindrical and cuboid nanostructures, which enable lower fabrication complexity, thereby obtaining RGB achromatic images (633, 532, and 488 nm)^108^.

Dispersion engineering has been exploited with a metasurface-integrated hyperspectral imager (HSI), which records spectral data for whole images (Fig. 11g)^193^. Conventional HSI encounters challenges related to compactness^203^, low-throughput^204^, and heavy postprocessing^205^. A metasurface-integrated HSI system comprises three reflective and one transmissive metasurface fabricated by a single lithography step on a glass substrate. The input aperture has been placed in one gold mirror, and metasurfaces have been placed in another. After entering the light from an object through an aperture, the light is vertically dispersed by a reflective lens, whose functionalities are similar to the first-order blazed grating. The other two reflective and one transmissive lenses focus light into a 2D array detector. Finally, the light is dispersed in the horizontal direction along the incident angle and vertically along the wavelength after passing the HSI in the transmissive metasurface. In addition, these metasurfaces have been optimized by ray tracing and particle swarm optimization for the desired wavelengths (750–850 nm) and spatial range ($\pm \!15^\circ$).

Multilayered metasurfaces have been used to facilitate tunable information encryption both optically and physically. For example, shareholder metasurfaces have been designed, and they show various images when physically cascaded (Fig. 11h)^206^. If every shareholder is collected, a secret share is observed, which is independent of the information of each holder^207^. The secret image of the cascaded holographic metasurface is divided into two phase profiles via computational division (encryption process), representing two independent images pixel-wise. Each holder shows an independent holographic image. However, the combination of holders 100 nm apart reveals the secret image (decryption process). Furthermore, tunable hologram images are demonstrated by encoding multiple images across relative translational positions of two cascaded metasurfaces.

Cascaded metasurfaces have also been applied to Moiré metalenses, which provide a wide tunable focal length with respect to the mutual rotation angle between two metasurfaces (Fig. 11i)^208^. Moiré metalenses have been demonstrated at a NIR wavelength (900 nm) with insensitive polarization meta-atoms, which comprise amorphous silicon for a high index contrast, and they achieve the maximum NA of 0.5. It also shows a range of focal length tunability between ±1.73 and ±5 mm along the mutual rotation angle of ±90°. The fundamentals of Moiré metalenses lie in asymmetric phase distribution, and their combination of rotation becomes a symmetric lens phase distribution. Positive rotation angles make the total phase combination of the two layers resemble a convex Fresnel lens, whereas negative angles exhibit a concave-like distribution. Moreover, an increase in the rotation angle produces a higher phase gradient, resulting in a shorter focal length and higher optical power.

## Perspective on integrated metasurfaces for the near-future applications

Recently, wearable displays (VR/AR), LiDAR, and bio/chemical sensing have received considerable attention as practical applications of metasurfaces. Although various optical materials and devices have been developed in lab-scale experiments with free-from optics^161,209^, it requires many complex steps and efforts to result in actual commercialization. However, metasurfaces have continuously offered many advantages, such as compact size, wide FoV, and molecular level sensitivity, and they have steadily gained great interest in the industry fields of VR/AR, LIDAR, and bio/chemical sensing. Herein, we consider the function and achievement of a metasurface in a large application system.

### Wearable display system (VR/AR)

VR and AR are crucial wearable display technologies for the metaverse. VR technology replaces the real world with virtual images, thereby providing users with an immersive experience^210^. AR integrates computer-generated three-dimensional (3D) images into the real world. Despite the rapid development of related technology, VR/AR technology has suffered from several problems such as chromatic aberration, narrow FoV, and spherical aberration, resulting in a large form factor. Consequently, current devices in the market are bulky and have performance flaws.

Recently, metasurfaces have been widely used to construct compact VR/AR systems. Metasurfaces have been attached to contact lenses for a near-eye display, and metasurfaces with spatially encoded phase maps project virtual information using a pixel-wise method^211^. See-through anisotropic metalenses correct chromatic aberration with a wide FoV^212^. These metalenses use both handednesses of the circular polarization state to achieve a see-through mode. However, they work only at green wavelengths with a low-quality hologram^211^ and require additional optical components such as dichroic mirrors and circular polarizers^212^.

**Fig. 12:**
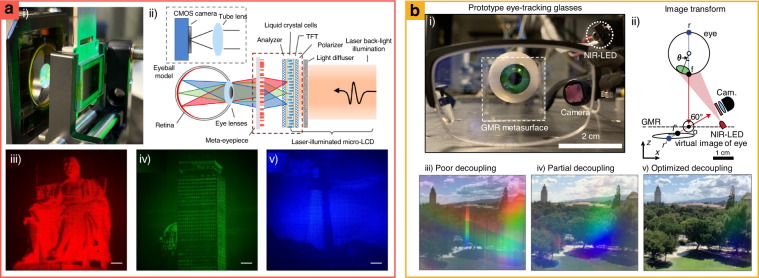
Metasurface-integrated wearable display system for (a) Virtual reality (VR) and (b) augmented reality (AR). **a** VR with cm-scale RGB achromatic metalens. **a.i** set-up image and **a.ii** its schematics of VR system. Grayscale VR images with **a.iii** red, **a.iv** green, and **a.v** blue. **b** AR with eye-tracking supporting metasurfaces. **b.i** Actual configuration and **b.ii** schematic of the system. Scattered near-infrared light from the eye is reflected by GMR metasurfaces, and then captured by the camera. **b.iii** Poor decoupling is served in antireflection coating glass surface with a strong rainbow. **b.iv** Partial decoupling in GMR metasurfaces with 7-nm-thick p-Si grating. **b.v** Optimized decoupling in GMR metasurface with 3-nm-thick p-Si grating. **a** is reproduced with permission from ref. ^213^ Copyright © 2022, Zhaoyi Li et al., **b** from ref. ^217^ Copyright © 2021 Springer Nature

One promising miniaturization approach is to reduce the number of optical components by polarization-insensitive achromatic metalenses. For example, large-scale achromatic metalenses have been used as near-eye optical components in VR imaging systems (Fig. 12a)^213^. The achromatic metalens-integrated VR devices consist of a laser-illuminated micro-LC display (µLCD), an eyeball model, and a meta-eyepiece. The meta-eyepiece corresponds to an achromatic metalens with polarization-insensitive properties and a diameter of 1 cm. Consequently, compact optical systems have been realized with achromatic metalenses showing grayscale VR images at three wavelengths with arbitrary polarization states from the µLCD.

Moreover, metasurfaces have demonstrated the feasibility of wearable AR glass. Multiplexing hologram images have been demonstrated by metasurface hybridized with waveguides in AR projection systems^214,215^. Further, Huygens’ metasurfaces demonstrate a continuous view of a 3D hologram in a near-eye display to solve vergence-accommodation conflicts with large pixel counts and subwavelength pixels^216^. Coupled with these technologies, eye-tracking in non-local metasurfaces can realize the real application of AR glass.

An eye-tracking system that uses reflected light from the faces of glasses has suffered from performance degradation by the limitation of decoupling between visible wavelengths for the real world and NIR for tracking. To solve this problem, non-local metasurfaces have been applied in eye-tracking technology, facilitating a low-rainbow background and high transparency (Fig. 12b)^217^. Metasurfaces for AR systems have been designed with guided-mode resonators (GMRs)^218–220^ and a high-*Q* factor that selectively reflects NIR wavelength for tracking. Polycrystalline Si (p-Si) strips with spectral dependency on the absorption depth are placed on the dielectric waveguide. In the visible spectral range, the low-*Q* factor light has dominant optical losses in poly-Si structures, which suppresses the undesired diffraction. Consequently, visible light almost passes through the metasurface without a rainbow effect while scattering at *λ* = 870 nm, where the metasurfaces exhibit resonance. Further, the NIR light operates a large internal electric field, enabling diffraction at the desired angle while being trapped and sufficiently guided in the waveguide, resulting in the reflection with over 10% efficiency of the first diffraction order. When the NIR LED illuminates the eye, the metasurface reflects the NIR light from the eye to the side camera which captures the front view of the eye image to analyze the motion of the eye.

### High-performance LiDAR

LiDAR is a depth-scanning technology that determines the distance by analyzing the reflected light from target objects used for autonomous vehicles, unmanned aerial vehicles, or intelligent robots. Two representative depth scanning methods have been used for LiDAR: (1) indirect^221,222^ or direct-ToF, and (2) structured light (SL). The direct ToF technique estimates the distance by analyzing the round-trip flight time *t*_laser_ of the laser pulse, and the distance is then calculated as *ct*_laser_/2, where *c* is the speed of light. The ToF technique is generally classified into scanning or non-scanning ToF system; The classical scanning ToF suffers from the trade-off between framerate and FoV owing to the inertia of its mechanical moving component. Non-scanning ToF illuminates the entire scene and measures the flight time from multiple points of the scene in a single shot. Since the power of the incident laser is divided by the number of illumination points, it requires a highly-sensitive photodetector, such as a single-photon-avalanche diode, in a 2D array, to acquire sufficient working distance. In the SL imaging technique, light is spread into predefined patterns (e.g., an array of dots, and lines) over a large FoV, and the surface profile of the object is calculated by analyzing distorted light patterns using a single camera or capturing structured light at different viewpoint using stereo cameras^223^. However, it suffers from low diffraction especially at large angles because of the large pixel size of conventionally used SL projectors (e.g., DOE, and SLM). Although there is an increasing demand for high-quality LiDAR systems, they face many challenges with conventional ROEs or DOEs, such as bulkiness, vulnerability to external impact, low scanning speed, and narrow FoV. Most recently, metasurfaces have improved these problems through incorporation within a LiDAR system^221,224,225^. Herein, we briefly introduce a metasurface-integrated LiDAR application in terms of scanning the ToF, and SL with a stereo camera.

**Fig. 13:**
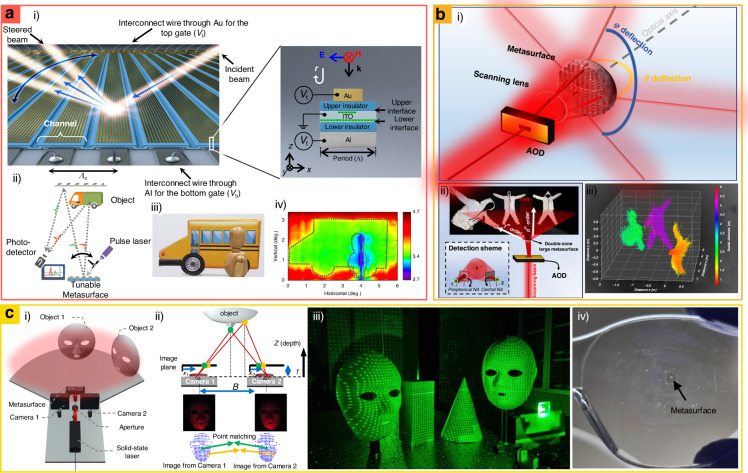
Metasurface-integrated light detection and ranging (LiDAR) with (a) electrically-tunable metasurfaces, (b) beam-steerers, and (c) point-cloud. **a** Electrically tunable metasurface-integrated LiDAR. **a.i** Electrically tunable metasurface reflects the light in varied directions depending on applied voltages, and cross-sectional view of meta-atoms of electrically tunable metasurfaces, which include two insulators and voltage gates. **a.ii** Schematic of the electrically tunable metasurface-integrated LiDAR system. This system detects the depth of objects in the middle image and the calculated depth data based on the ToF technique. **a.iii** Target objects and **a.iv** measured depth profile. **b** Metasurface-integrated acousto-optic deflector (AOD). **b.i** Schematic of fast active scattering system with metasurface-integrated AOD. **b.ii** Schematic of two strategies of depth reconstruction. **b.iii** Scanned depth information of human motion. **c** Point cloud metasurface-based depth sensor. **c.i** Schematic of point cloud metasurface-integrated SL system. **c.ii** Depth calculation method of stereo matching algorithm. **c.iii** Experimental demonstration of point cloud metasurfaces, which diffract high-density dot arrays over 180° field of view. **c.iv** Fabricated metasurface on curved surfaces of glasses by nanoimprint lithography. **a** is reproduced with permission from ref. ^224^ Copyright © 2021 Springer Nature, **b** from ref. ^225^ Copyright © 2022, Renato Juliano Martins et al., **c** from ref. ^230^ Copyright © 2022, Gyeongtae Kim et al.

Electrically tunable metasurfaces have been implanted with ToF scanning-type LiDAR system by exploiting their fast manipulation of wavefront^224^. To improve the LiDAR performance, a new electrical SLM is connected to the metasurface without conventional devices, such as LCs, and MEMS, which limits the reliability and speed. Electrically tunable metasurfaces for LiDAR have been designed with an Au nanoantenna, Al mirror as independent voltage gates, and ITO layer as ground; between the ITO and metallic layers, an oxide insulator is placed (Fig. 13a). The combination of each metallic gate voltage changes the reflectance coefficient by varying the charge accumulation/depletion layer at the interface between the ITO and insulator layers. Consequently, the varied reflectance coefficients cover the full 2${\rm{\pi }}$ phases with an independent amplitude in the metasurface, where the ITO layer is exceedingly thin for a single gate to cover the full phase^124,226,227^. Electrically tunable metasurfaces successfully control the steering angle. Through the adoption of a receiver with electrically tunable metasurfaces, the depth profile has been estimated by measuring the light flight time. This ToF system achieves a switching speed of 5.4 MHz, which is sufficient for scanning in commercial LiDAR systems; however, it has a limited FoV of 6° × 4° with a diffraction efficiency of 1% for full phase control in the metasurface alone.

Another LiDAR method, metasurface-integrated acousto-optic deflector (AOD) has been also developed to enlarge the limited FoV^225^. It simultaneously creates a large FoV and high speed without compromising the framerates or large FoV (Fig. 13b)^225^. The AOD can rapidly repoint an incident laser at an arbitrary angle; however, it has a small FoV (2° × 2°) which is not sufficient for application in LiDAR. Its small FoV is enlarged to 150° × 150° by integrating metasurfaces with AOD, and the metasurface-integrated AOD also achieved high-speed alteration of the deflection angle up to 250 MHz. In addition, the additional AOD achieves two axial scans for 3D imaging. When the metasurface-integrated AOD is used to recognize and reconstruct a human motion, it can simultaneously detect multizone images with the peripheral (low resolution) and fovea regions (high resolution) using an irradicable zeroth-order beam from metasurfaces. Consequently, this system can mimic the human vision system and be applied to advanced driver assistance systems (ADASs).

A wide FoV has also been demonstrated with metasurface-integrated SL imaging systems^228–230^ where electrical scanning control is no longer needed for close-range scans. Recently, metasurfaces have been designed as point cloud spreaders to create a uniform dot-array form with a high density of ~10K dot at a wide FoV^230^. They are then used for SL imaging systems with two cameras for point capturing (Fig. 13c). After spreading the point cloud with metasurfaces, a stereo system reconstructs the 3D depth of the object by capturing the scattered dot arrays on the object surface using two cameras at different view angles. The points of two camera images are matched through comparison^231^, and the depth is calculated using stereo camera trigonometry with the location coordinates of matched pairs. If the metasurface is integrated with commercial glasses using nanoimprinting, it is expected to overcome the limitation of conventional bulky systems that require mechanical rotators, which decreases the framerate and robustness to external impact^232,233^.

### Ultrasensitive bio/chemical sensors

Optical sensors infer the characteristics of a target by analyzing scattered or absorbed light. Label-free biochemical detection is an important technique that does not modify molecules with fluorescent or radioactive dyes. Thus, this technique, when applied to bio-analytics or diagnostics, is inherently noninvasive and highly sensitive. When a non-local metasurface with the q-BIC mode is applied to a biochemical sensor, it provides label-free detection via highly surface-sensitive resonance, enabling high sensitivity and chemical specificity. The metasurface can also provide compactness to the sensing system by realizing a spectrometer-less system. In this review, we investigate biochemical sensors that offer the advantages of metasurfaces.

**Fig. 14:**
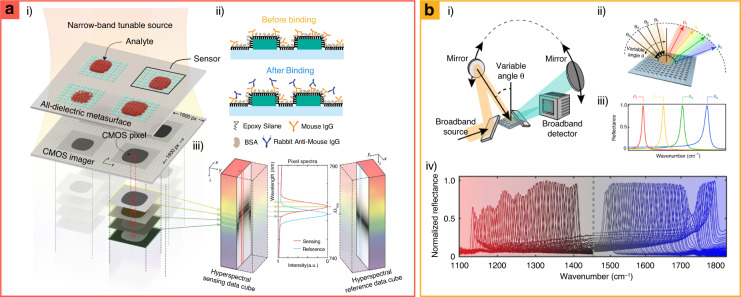
Metasurface-integrated bio/chemical sensors for (a) quantitative and (b) qualitative analyses. **a** Non-local metasurface integrated hyperspectral imaging (HSI). **a.i** Schematics of the system. Each sensor is detected by tens of thousands of CMOS pixels. **a.ii** Schematic of bioassay where epoxy-silane immobilizes the mouse IgG, binding rabbit anti-mouse IgG. Bovine serum albumin (BSA) is deposited to control the areal molecular density by combining with epoxy instead of IgG. **a.iii** Resonant peak profile is compared with the reference profile where each profile is produced by sweeping the wavelength without or with the analyte. **b** Metasurface-integrated angle-multiplexed sensors. **b.i** Schematic of the system. **b.ii** Angle-multiplexed metasurface exhibiting different resonance wavenumbers along the incidence angle. **b.iii** Different resonance peaks along an incident angle. **b.iv** Normalized reflectance spectra after coating analyte that can be recognized by analyzing reflectance patterns. **a** is reproduced with permission from Ref. ^234^ Copyright © 2019 Springer Nature, **b** from ref. ^237^ Copyright © 2019 The Authors, some rights reserved; exclusive licensee American Association for the Advancement of Science, Distributed under a CC BY-NC 4.0 license http://creativecommons.org/licenses/by-nc/4.0/, Reprinted with permission from AAAS

An HSI system has been demonstrated with non-local metasurfaces for tracing biomolecules (Fig. 14a)^234^. Non-local metasurfaces confine electric fields on the surface of meta-atoms, enabling extreme responsibility for the local refractive index change from the spatial overlap of individual biomolecules^235,236^. When biomolecules are placed on the sensor, a change in the local refractive index shifts the resonance peak along the quantitative value of the bio-samples. The system’s sensing performance has been verified using a biorecognition assay with immunoglobulin G (IgG). It has been found to achieve a higher molecule per area sensitivity compared to that of the conventional ensemble-average method.

The angle-multiplexed resonance of q-BIC metasurfaces has been exploited to detect the molecular absorption signature associated with the vibrational mode and absorption band of each molecular chemical bond, thereby enabling molecular categorization (Fig. 14b)^237^. Angle-multiplexed q-BIC metasurfaces are designed to exhibit continuous resonance wavevector that changes along the incidence angle with asymmetric structure in the *x*-axis, and the resonance wavevector covers the range of 1080–1820 cm^−1^. While illuminating light at variable incidence angles with a moving mirror and spectrometer, molecules adsorbed on metasurfaces cause attenuation of resonance line shape by making near-field coupling. Consequently, the absorbance spectrum of high sensitivity and spectral selectivity is characterized by diverse molecules. The absorption spectrum of thin polymethyl methacrylate (PMMA) has been demonstrated by obtaining a result similar to that of standard IR reflection absorption spectroscopy (IRRAS). The bioassay process to detect human odontogenic ameloblast-associated protein (ODAM) with a spectrometer-less sensor, including a broadband source, has shown a detection limit of 3000 molecules per µm^2^ and a surface mass sensitivity of 0.27 pg mm^−2^.

## Conclusions

In summary, metasurface-based optical systems have accomplished great success by presenting high-resolution receivers, polarization-controlled single-photon emitters, and tunable wavefront controllers. Also, by combining classical optical components, the performance of planar waveguides, optical fibers, and ROEs has been extended. Composed metasurfaces have provided spatial wavefront control^199^, high-end optical security^195^, and polarization analyzer^200^. In the perspective section, this review provides the future direction of metasurface-integrated photonic applications with examples of recent works including VR/AR, LiDAR, and sensors.

However, from our perspectives, three challenges have remained for the commercialization of metasurfaces-based optical systems. In general, metasurfaces are mostly manufactured with the CMOS process, and many researchers argue that the CMOS process is advantageous to commercialize when metasurfaces are implanted for commercial devices. However, it is generally not feasible with optic module manufacturing processes such as injection molding or milling. Also, the CMOS process is a higher-cost production method than that of ROEs and DOEs, increasing the total production costs of a metasurface-integrated optical platform. Another challenge is related to the low efficiencies of metasurfaces. Although metasurface efficiencies reach over 90% at a single wavelength, achromatic metalenses at visible^238^ still have lower efficiencies (40%) than those of conventional ROEs (>95%, Thorlabs, mounted achromatic doublet). Since the efficiencies of the total optical system cannot exceed the minimum efficiency of its optical components, the use of metasurfaces is not suitable for applications requiring efficient light manipulation. The other challenge lies in the quantification methods of metasurfaces. Compared to conventional ROEs and DOEs, the metasurface quantification method has not been unified yet^7^. For example, in the case of metalenses, various groups define different definitions of efficiencies and use different measurement systems^7^. The different figure of merits prohibits them to be compared with not only metasurfaces but also other conventional optical systems when they are integrated with metasurfaces.

Regardless of these challenges, we believe that metasurfaces will be essential components to designing future optical platforms such as detectors of automobile vehicles^221^, displays of wearable devices^54^, and healthcare monitors for precise diagnostics^239^. Nanofabrication methods have been developed to be more compatible with advanced optical materials, and their efficiencies have continuously increased with the bandgap engineering of optical materials. For example, particle-embedded resin^240–243^ and large-area, low-loss dielectric deposition methods^244–248^ have been recently demonstrated only for metasurface manufacturing, achieving low-cost production of large-area metasurfaces with near-unity efficiencies. These methods enable the cost-effective process of metasurface manufacturing, and its production cost will be compatible with conventional ROEs and DOEs. Moreover, considering most recent reports have proposed unified quantification methods of metasurfaces, we believe that metasurface-integrated optical devices will be a promising option to construct near-future photonic platforms, enabling a broad range of applications for metasurface-integrated photonics in everyday life.

It has been already proven that metasurface offers an effective and feasible way to engineer electromagnetic waves, while there is increasing demand for compressing sizes and extremely manipulating light. To bring metasurfaces into real-life devices beyond the current prototypes, many approaches should be conducted by integrating metasurfaces with transitional components rather than competing with conventional optical systems.
